# Mental health of Ukrainian refugees in Germany compared to other population groups – Results from the IAB-BAMF-SOEP Survey 2023

**DOI:** 10.25646/14267

**Published:** 2026-07-01

**Authors:** Susanne Bartig, Stefan Liebig, Carmen Koschollek, Adriana Rocío Cardozo Silva, Louise Biddle

**Affiliations:** 1 Freie Universität Berlin, Department of Sociology, Berlin, Germany; 2 Robert Koch Institute, Department of Epidemiology and Health Monitoring, Berlin, Germany; 3 German Institute for Economic Research (DIW), Socio-Economic Panel, Berlin, Germany; 4 Hochschule Hannover (HsH), University of Applied Sciences and Arts, Hannover, Germany

**Keywords:** Depressive symptoms, Anxiety symptoms, Mental health, Refugees, Migration, Social determinants, Health inequalities, Discrimination, Housing satisfaction, Social integration, Employment, Language, Ukraine, Germany, SOEP

## Abstract

**Background:**

To date, little is known about the mental health of Ukrainian refugees living in Germany. Therefore, this study aims to describe the prevalence of depressive (DS) and anxiety symptoms (AS) among refugees from Ukraine, identify relevant associated factors, and compare the prevalence of psychological distress across different groups of origin.

**Methods:**

The analyses are based on data from the IAB-BAMF-SOEP Survey of Refugees (2023), supplemented by data from the SOEP main survey. Age-adjusted, weighted prevalences of psychological distress were calculated for non-migrants (n =13,210), non-refugee migrants (n = 4,889), as well as refugees from Ukraine (n = 3,039) and those from Afghanistan, Iran, Iraq, and Syria (n = 3,011). To investigate associations between psychological distress and selected social determinants, prevalence ratios were estimated using Poisson regressions.

**Results:**

The age-adjusted prevalences of DS (21 %) and AS (13 %) were higher among refugees from Ukraine than among non-migrants and non-refugee migrants. Overall, women reported psychological distress more frequently than men, regardless of experiences of migration or forced displacement. Social integration, experiences of discrimination, and employment were important factors associated with mental health among refugees from all countries of origin included in the study. Among Ukrainians, associations with age, gender, income, and housing satisfaction were also evident, whereas German language proficiency was associated with mental health among refugees from other countries of origin.

**Conclusions:**

The results indicate a substantial need for psychosocial support services. Furthermore, post-migration factors were closely associated with psychological distress, with experiences of exclusion and discrimination in particular being linked to poorer mental health.

**Key messages**

► Refugees from Ukraine report depressive symptoms more frequently than non-migrants and non-refugee migrants.
► The prevalence of anxiety symptoms is higher among refugees from all countries of origin considered in this study than among non-migrants.
► Social integration is an important determinant of mental health, particularly among refugees from Ukraine.
► Experiences of discrimination are associated with symptoms of depression and anxiety among refugees, regardless of their country of origin.
► Among refugees from Ukraine, age, gender, income, employment status, and housing satisfaction are also associated with mental health.

## 1. Introduction

Russia’s war of aggression against Ukraine in February 2022 triggered the largest forced discplacement in Europe since the end of the Second World War. By the end of 2025, more than four million people had fled Ukraine to countries of the European Union (EU); with more than one million seeking protection in Germany [1]. Although Ukrainians currently constitute the largest group of refugees in Germany [2], little is known about their mental health.

The mental health of people with experiences of migration or forced displacment is influenced by various factors before, during, and after migration [3, 4]. Health risks and opportunities also vary within migrant populations. Refugees, in particular, are exposed to health risks due to traumatic experiences and physical strain both before and during forced migration [5, 6]. Previous studies also highlight the significant role of living conditions in the host country for mental health (‘post-migration factors’), such as accommodation in shared housing [6, 7], uncertainties regarding residence status [6, 8], unemployment [8, 9], separation from family members [10], and experiences of discrimination [11]. A systematic review showed that the prevalence of depression and anxiety disorders is significantly higher among refugees than among individuals who migrate for work-related reasons [12].

However, it remains unclear to what extent the existing evidence on the mental health of refugees can be applied to those from Ukraine, as forced migration from Ukraine differs from previous refugee movements in several important respects. Differences are observed in both legal regulations and sociodemographic composition, with a high proportion of women among Ukrainian refugees. The visa-free entry of Ukrainian nationals into EU member states, as well as the geographical proximity to the main host countries, represent favorable structural conditions. In contrast, the escape routes of refugees from other countries of origin, such as Syria, are often characterized by complex trajectories involving significant dangers and high costs. Furthermore, Ukrainians are granted temporary protection without being required to undergo a lengthy asylum procedure. The ‘Temporary Protection Directive’ regulates access to healthcare, the labour market, and social security benefits [13]. Consequently, the Asylum Seekers Benefits Act (Asylbewerberleistungsgesetz, AsylbLG, in particular Sections 4 and 6) does not apply to refugees from Ukraine, but restricts access to healthcare and the scope of benefits for refugees from other countries of origin during the initial years of their stay in Germany [14].

Previous studies from European host countries indicate a high risk of mental disorders, such as depression or anxiety, among refugees from Ukraine [15–17]. However, the robustness of these findings is limited due to small sample sizes and restricted representativeness. Furthermore, there is a lack of comparative analyses of mental health with other population groups, such as migrants without experiences of forced displacement. The present study addresses these gaps by providing the first comparative analysis of the mental health of refugees from Ukraine living in Germany based on a nationally representative sample and by identifying relevant associated sociodemographic and post-migration factors. 

Accordingly, this study addresses the following research questions:

1. What is the prevalence of psychological distress (depressive and anxiety symptoms) among people living in Germany with and without experiences of migration and forced displacement, based on representative data?
2. Which sociodemographic and post-migration factors are associated with the mental health of refugees from Ukraine? Are there differences compared to refugees from Afghanistan, Iran, Iraq, and Syria?

## 2. Methods

### 2.1 Data

#### IAB-BAMF-SOEP Survey of Refugees

The IAB-BAMF-SOEP Survey of Refugees is a longitudinal panel survey that has been conducted annually since 2016 in cooperation between the Institute for Employment Research (IAB), the Socio-Economic Panel (SOEP) at the German Institute for Economic Research (DIW) Berlin, and the Research Center of the Federal Office for Migration and Refugees (BAMF-FZ) [18]. The survey aims to provide representative data on the living conditions of people who have sought protection in Germany since 2013. To account for changes in refugee migration over time, refreshment samples are drawn regularly. Since 2023, the study has also included a separate sample of refugees from Ukraine (SUARE) [19]. In addition, a refreshment sample of refugees who had arrived in Germany from other countries of origin between January 2017 and August 2022 was drawn in 2023 [20]. For the present study, data from the 2023 wave of the IAB-BAMF-SOEP Survey of Refugees were analysed.

The sample of Ukrainian households for 2023 consists of two subsamples. The first sample (SUARE 1) comprises refugees who moved to Germany from Ukraine between 24 February and 8 June 2022, and who have already participated in the study ‘IAB-BiB/FReDA-BAMF-SOEP – Refugees from Ukraine in Germany’ [21]. The second sample (SUARE 2) is a refreshment sample and includes individuals with Ukrainian citizenship who arrived in Germany between 9 June and 31 August 2022. The random sample was drawn from the Central Register of Foreign Nationals (‘Ausländerzentralregister’, AZR) [20].

Data collection was conducted between July 2023 and January 2024 using a mixed-mode design, with computer-assisted personal interview (CAPI) as the primary mode. In addition to multilingual face-to-face interviews, self-administered survey modes were available: The questionnaire could be completed either on a tablet while another household member was being interviewed (CASI) or online at a later time (CAWI). Respondents could also participate in various languages (Arabic, German, English, Farsi, Russian, Turkish, and Ukrainian). In addition to sociodemographic and socioeconomic information (e.g., educational qualifications and their recognition, employment status, income), the survey collected data on language proficiency and language course attendance, intentions to stay, housing and family situation, life satisfaction, discrimination, health, and the nature and extent of social contacts. The study design and sample are described in detail elsewhere [20, 22]. 

Households were contacted in advance by the survey institute, with the gross sample comprising 4,715 (SUARE 1) and 6,720 (SUARE 2) households, respectively [19]. A total of 2,381 Ukrainian households participated in the survey, resulting in 3,662 individual interviews with adult household members [19]. According to the standards of the *American Association for Public Opinion Research* (AAPOR), the response rate (RR2) at the household level was 39.1 % (SUARE 1) and 15.1 % (SUARE 2) [20]. 

To provide a comparative analysis of the mental health of people living in Germany with and without experiencies of migration or/and forced displacement, the analyses were supplemented with data from the SOEP main survey (SOEP-Core). Thus, the IAB-BAMF-SOEP Survey of Refugees is integrated into the long-established SOEP [23], a nationally representative longitudinal study of private households in Germany, which currently includes approximately 30,000 individuals from about 20,000 households. Data collection took place between August and December 2023, also using a mixed-mode design. Detailed information on the sample, the study design, and the response rates of the SOEP-Core has been published in the corresponding methodological report [22].

### 2.2 Indicators

#### Psychological distress

To describe psychological distress, the presence of depressive and anxiety symptoms was selected, as these are widespread in the population and are among the most common mental health consequences of forced displacement and migration. In 2023, psychological distress was assessed in both SOEP-Core and IAB-BAMF-SOEP Survey of Refugees, using the established *Patient* *Health* *Questionnaire-* *4* (PHQ-4) [24]. The PHQ-4 is a reliable and valid brief screening instrument for assessing depressive and anxiety symptoms in population-based surveys [25]. Using two items each, this instrument measures the core symptoms of depression (PHQ-2: ‘little interest or pleasure in doing things’, ‘feeling down, depressed, or hopeless’) and generalized anxiety disorder (GAD-2: ‘feeling nervous, anxious, or on edge’, ‘unable to stop or control worrying’) based on participants’ self-reports in a questionnaire. Participants rate the occurrence of these symptoms over the past two weeks on a 4-point Likert scale with ‘not at all’ (0), ‘on a few days days’ (1), ‘on more than half of the days’ (2), and ‘(almost) every day’ (3). A score of 3 or higher on the respective scale was used as the cut-off indicating depressive or anxiety symptoms [26]. Based on the respective cut-off values, dichotomized variables were generated for depressive and anxiety symptoms, distinguishing participants with symptoms from those without symptoms.

#### Comparison groups

The SOEP collects information on the respondents’ country of birth as well as the country of birth and nationality of their (grand)parents to capture individual and family migration histories [27]. Additionally, it takes into account whether the respondents migrated to Germany in the context of forced displacement. For the comparative analysis of psychological distress, participants were classified into the following groups based on these measures:

1. People without a history of migration or forced displacement, i.e., the person themselves and both parents were born in Germany (hereinafter referred to as non-migrants)
2. People who immigrated to Germany on their own but not in the context of forced displacement (hereinafter referred to as non-refugee migrants)
3. Refugees from Ukraine who arrived in Germany between 24 February and 31 August 2022, and
4. Refugees who arrived in Germany from Afghanistan, Iran, Iraq, and Syria between 2013 and 2023

Due to small sample sizes, people with parental migration experience – i.e., individuals born in Germany with at least one foreign-born parent – were not included in the analyses.

#### Sociodemographic and post-migration factors

The selection of sociodemographic and post-migration factors was based on existing research on the mental health of refugees. In addition to gender, the age of the respondents was taken into account in the analyses and categorized into the groups ‘18 to 39 years’, ‘40 to 59 years’, and ‘60 years and older’. Based on the participants’ educational and vocational qualifications, education was classified according to the 2011 version of the *International Standard Classification of Education* (ISCED 2011 [28]) into ‘low’ (ISCED 0 – 2), ‘medium’ (ISCED 3 – 4), and ‘high’ (ISCED 5 – 8) groups. Net equivalence income was calculated using participants’ information of their households’ monthly net income and adjusted for household composition, taking into account the number and age structure of household members using the revised equivalence scale of the Organization for Economic Co-operation and Development (OECD) [29]. For the analyses, the weighted median of equivalized income was calculated, and the low-income threshold was defined as 60 % of this median. Individuals with an income below this threshold were classified as low-income earners. In addition, employment status was included in the analyses and dichotomized into ‘employed’ and ‘unemployed’.

Based on information regarding current marital or partnership status and the corresponding place of residence, the ‘partnership’ variable was categorized as follows: ‘single’, ‘partner in Germany’, and ‘partner abroad’. To assess participants’ social contacts, the frequency of ‘visiting or being visited by neighbors, friends, or acquaintances’ was used. The response options ‘daily’ and ‘at least once per week’ were combined and compared with those who reported ‘at least once per month’, ‘seldom’, or ‘never’. 

In addition, self-reported experiences of discrimination were included in the analyses; these were defined as being treated less favorably ‘for example, due to your ethnic origin, racial reasons, gender, religion or beliefs, disability, age, or sexual orientation’. Participants were asked whether they had felt discriminated against in the past 12 months in various areas of life - including ‘job’, ‘hairdressers, bars, restaurants, and similar places’, ‘looking for an apartment or a house’, ‘health or care sector’, ‘public offices or authorities’, and ‘public transport’. Participants who answered ‘yes’ in at least one of the 12 areas were compared with those who reported no experiences of discrimination. 

Since refugees from other countries of origin have lived in Germany longer on average, the type of accomodation (collective accomodation vs. private household) was not included in the analyses; instead, housing satisfaction was considered. Participants rated their overall satisfaction with their current housing situation on a scale from 0 (‘completely dissatisfied’) to 10 (‘completely satisfied’). For the analyses, the responses were categorized into ‘low’ (0 – 3), ‘medium’ (4 – 6), and ‘high’ (7 –10). 

To describe German language proficiency, an index was created based on respondents’ self-assessment of their German reading, writing, and speaking skills on a scale ranging from 1 (‘very good’) to 5 (‘not at all’). To improve interpretability, the items were recoded so that higher values reflect better language proficiency. The summary index combines the recoded self-assessments into a measure of general German language proficiency and was categorized as ‘good’, ‘moderate’, and ‘poor’. 

### 2.3 Statistical methods

To address the first research question, age-adjusted prevalences of depressive (PHQ-2) and anxiety symptoms (GAD-2) were calculated to account for the differences in the age distributions of the selected comparison groups. Age adjustment was performed using predictive margins from survey-weighted logistic regression models. These are model-based prevalence estimates that were standardized according to the age distribution of the total sample, thereby enabling comparisons between groups independent of differences in age structure.

In addition, prevalence ratios (PR) were calculated using Poisson regressions to investigate the associations of sociodemographic and post-migration factors with psychological distress (Research Question 2). The multivariable analyses focus on a comparison between refugees from Ukraine and those from Afghanistan, Iran, Iraq, and Syria, as not all of the included variables were part of the SOEP main survey (SOEP-Core). The regression analyses controlled for the degree of urbanization (urban vs. rural), East and West Germany, and length of stay. The PRs and their corresponding 95 % confidence intervals (95 % CI) are presented in the results as forest plots. If the respective 95 % CI does not include the value 1, an association between the selected factor and psychological distress can be assumed. PRs above 1 indicate a higher (below 1 a lower) prevalence of depressive or anxiety symptoms in the group compared to the reference group. Due to the unequal gender distribution within the comparison groups, gender-stratified sensitivity analyses were also conducted. This ensured that the associations identified in the overall model were not attributable to the unequal gender composition in the subsamples. 

A weighting factor was included in the analyses to ensure representativeness of the target populations and to account for differences in selection and participation probabilities. The survey weight consists of both a design weight accounting for response probabilities and a calibration adjustment. For the IAB-BAMF-SOEP Survey of Refugees, the adjustment is performed using information from the AZR on age, gender, federal state, and date of arrival (year and quarter) [19]. For SOEP-Core, the margin adjustment is performed using data from the Microcensus on age, gender, nationality, federal state, municipality size class, home ownership, and household size [30]. For participants who already took part in the study in the previous year, the previous wave weight is also taken into account.

All analyses were performed using Stata/SE 19.0 (Stata Corp., College Station, TX, USA, 2019) with survey procedures for complex samples.

## 3. Results

### 3.1 Sample description

Table 1 shows the sociodemographic and socioeconomic characteristics in the study population. Of the 3,039 Ukrainians included in the study, nearly three-quarter were female (73.1 %), indicating substantial differences in gender distribution relative to the selected comparison groups. Whereas approximately half of the non-migrants (51.1 %) and non-refugee migrants (53.0 %) were female, the proportion was only one-third (33.7 %) of refugees from Afghanistan, Iran, Iraq, and Syria. Furthermore, differences in age distribution were observed across the groups. The median age among refugees from Ukraine was around 40 years. Accordingly, they were older than refugees from Afghanistan, Iran, Iraq, and Syria (median age 33 years), but younger than non-migrants (median age 56 years) and non-refugee migrants (median age 44 years). Regarding the socioeconomic characteristics of Ukrainians, 60.3 % belonged to the high ecudation group and 30.7 % to the middle education group, indicating a higher level of educational attainment than the comparison groups. Furthermore, there were substantial income disparities within the study population. While the weighted median of monthly equivalised net income among refugees from Ukraine was around 927 euros, it was nearly 2,200 euros among non-migrants and around 2,000 euros among non-refugee migrants. Refugees from other countries of origin had a comparable income level to those from Ukraine with 975 euros. Accordingly, the proportion of low-income individuals was higher among refugees from Ukraine (85.0 %) than among non-migrants (12.7 %) and non-refugee migrants (22.7 %). Given their shorter length of stay in Germany, the proportion of individuals who were not employed was also higher among refugees from Ukraine than among non-refugee migrants and non-migrants. 

With regard to post-migration factors, more than one-third (38.7 %) of Ukrainians were single, whereas nearly half (46.1 %) reported having a partner living in Germany (Supplementary material Table 1). In contrast, 15.2 % of refugees from Ukraine and 8.5 % of those from Afghanistan, Iran, Iraq, and Syria stated that their partner was living abroad. More than half of the refugees from Ukraine (55.8 %) and from the other countries of origin considered in this study (51.7 %) reported visiting neighbors, friends, or acquaintances at least once a week. Experiences of discrimination were reported by more than one-third of refugees from Ukraine (35.1 %) and from the other countries of origin (38.7 %). The vast majority of Ukrainians were very satisfied with their current housing situation (71.7 %); only 8.5 % reported low housing satisfaction. In contrast, refugees from Afghanistan, Iran, Iraq, and Syria exhibited lower levels of housing satisfaction (high: 56.7 %, low: 17.0 %). Nearly half of Ukrainians (44.5 %) rated their German language proficiency as poor, whereas 16.2 % reported a good level. Among refugees from the other selected countries of origin almost one-third (30.8 %) rated their German language proficiency as good.

### 3.2 Comparison of age-adjusted prevalences of psychological distress

**Figure 1:**
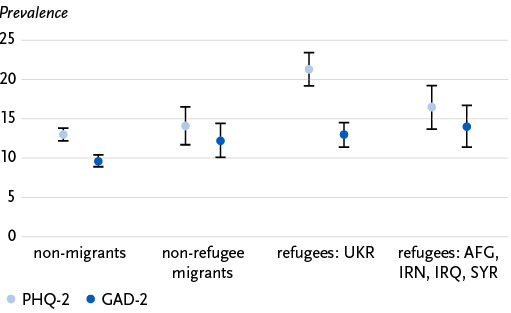
Age-adjusted prevalences of depressive (PHQ-2) and anxiety symptoms (GAD-2) (in %). Sources: IAB-BAMF-SOEP Survey of Refugees 2023, SOEP-Core 2023 UKR = Ukraine, AFG = Afghanistan, IRN = Iran, IRQ = Iraq, SYR = Syria

21.3 % of refugees from Ukraine reported depressive symptoms according to the PHQ-2 (95 % CI: 19.1 % – 23.5 %) (Figure 1; Supplementary material Table 2). Accordingly, they were more frequently affected by depressive symptoms than non-migrants (13.0 % (12.1 % –13.9 %)) and non-refugee migrants (14.1 % (11.6 % –16.6 %)). A comparison with the non-age-adjusted prevalence estimates revealed similar results (Supplementary material Table 3).

Refugees from Ukraine (13.0 % (11.3 % –14.6 %)) and refugees from the other countries of origin considered in this study (14.0 % (11.3 % –16.8 %)) exhibited higher prevalences of anxiety symptoms (GAD-2) than non-migrants (9.6 %; 95 % CI: 8.8 % –10.5 %). For both depressive and anxiety symptoms, age-adjusted prevalence estimates were consistently higher among women than among men across all comparison groups, regardless of experiences of migration or forced displacement (Supplementary material Table 4). While 22.3 % (19.9 % – 24.7 %) of Ukrainian women reported depressive symptoms, the proportion among Ukrainian men was 18.3 % (15.7 % – 20.9 %). A similar pattern was observed for anxiety symptoms, with the gender difference amounting to approximately 5 percentage points. Overall, gender differences were most pronounced for anxiety symptoms.

### 3.3 Associations of psychological distress with sociodemographic and post-migration factors

#### Depressive symptoms

**Figure 2:**
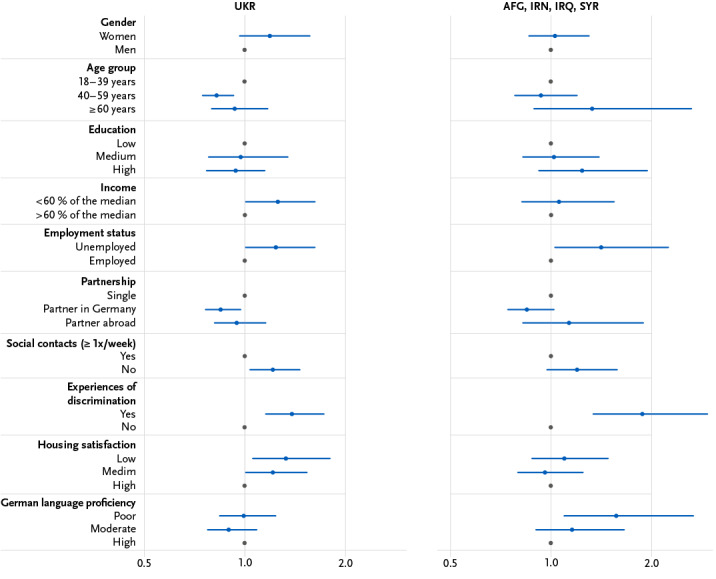
Determinants of depressive symptoms (PHQ-2) among refugees from Ukraine (n = 2,752) and those from Afghanistan, Iran, Iraq, and Syria (n =1,435). Results of Poisson regression models (prevalence ratios). Source: IAB-BAMF-SOEP Survey of Refugees 2023 UKR = Ukraine, AFG = Afghanistan, IRN = Iran, IRQ = Iraq, SYR = Syria

With regard to sociodemographic characteristics, the multivariable analyses showed that refugees from Ukraine aged 40 to 59 were less likely to report depressive symptoms than younger respondents (Figure 2, Supplementary material Table 5). Regarding income (< 60 % of the median), the multivariable results indicated that individuals from Ukraine below the low-income threshold had an increased likelihood of depressive symptoms. Employment status was relevant for both refugees from Ukraine and those from the other countries of origin considered in this study. Specifically, individuals who were not employed reported depressive symptoms more frequently than those who were employed.

While partnership status and the frequency of social contacts were not associated with depressive symptoms among refugees from Afghanistan, Iran, Iraq, and Syria, these post-migration factors were relevant among Ukrainians. For instance, individuals who reported exchanging visits (at least once a week) with neighbours, friends, or acquaintances had a lower likelihood of depressive symptoms than those without social integration. In addition, the results showed that individuals with a partner in Germany had a lower likelihood of depressive symptoms than those living alone. This association was not observed among refugees whose partner was living abroad.

Self-reported experiences of discrimination were associated with depressive symptoms among both refugees from Ukraine and those from the other countries of origin: Individuals who reported experiences of discrimination had a 1.5-fold and 1.9-fold higher prevalence of depressive symptoms, respectively, than those without such experiences. Furthermore, refugees from Ukraine with low or moderate housing satisfaction were more likely to report depressive symptoms than those with high satisfaction with their current housing situation. While German language proficiency was not associated with depressive symptoms among refugees from Ukraine, refugees from the other countries of origin with poor German language proficiency were 1.7 times more likely to report depressive symptoms compared with those stating good proficiency.

The gender-stratified sensitivity analyses (see Supplementary material Tables 6 and 7) revealed that associations were largely consistent with those observed in the overall models. In particular, the strong association between experiences of discrimination and depressive symptoms remained consistent across gender and countries of origin. Differences were observed primarily in the levels of statistical significance rather than in the overall interpretation of the findings. One exception was the association between depressive symptoms and low income (< 60 % of the median) among female refugees from Ukraine, which was in the opposite direction to that observed in the main model.

#### Anxiety symptoms

**Figure 3:**
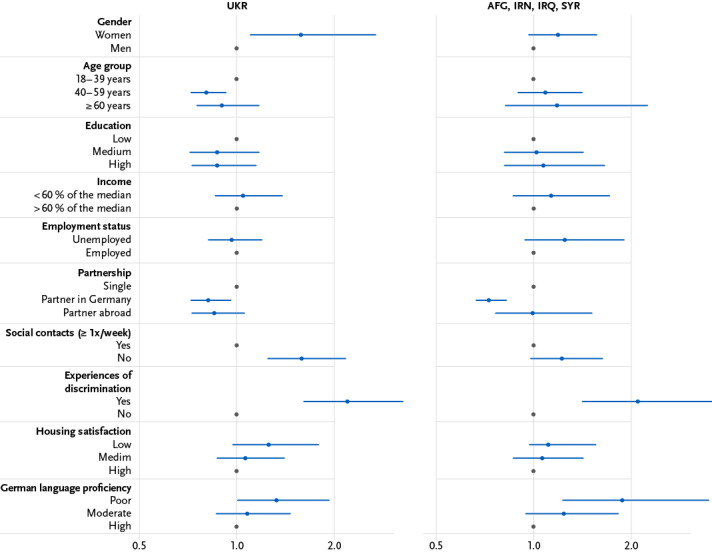
Determinants of anxiety symptoms (GAD-2) among refugees from Ukraine (n = 2,869) and those from Afghanistan, Iran, Iraq, and Syria (n =1,457). Results of Poisson regression models (prevalence ratios). Source: IAB-BAMF-SOEP Survey of Refugees 2023 UKR = Ukraine, AFG = Afghanistan, IRN = Iran, IRQ = Iraq, SYR = Syria

Among refugees from Ukraine, women were significantly more likely to report anxiety symptoms than men, and respondents aged 40 to 59 were less likely to report anxiety symptoms than those aged 18 to 39 (Figure 3, Supplementary material Table 8). Furthermore, refugees from Ukraine who reported infrequent social contact had a 1.7-fold higher prevalence of anxiety symptoms.

While income and employment were not associated with anxiety symptoms, partnership status was a significant determinant for both comparison groups. Specifically, the likelihood was lower among individuals whose partner was living in Germany than among those who were single. Regardless of country of origin, the prevalence of anxiety symptoms was approximately twice as high among individuals who reported experiences of discrimination as among those who had not experienced discrimination. Furthermore, German language proficiency was associated with anxiety symptoms in both groups, with refugees reporting poor German reading, writing, and speaking skills exhibiting a higher likelihood of anxiety symptoms than those reporting good proficiency.

The gender-stratified sensitivity analyses (Supplementary material Tables 9 and 10) confirmed the key findings of the overall models. In particular, experiences of discrimination were associated with an increased likelihood of anxiety symptoms among both women and men. At the same time, minor differences emerged with regard to individual determinants. For instance, social contacts were associated with a lower likelihood of anxiety symptoms among women from Ukraine, whereas stronger associations with German language proficiency were observed among men. However, the direction of the associations was consistent across both genders, indicating that the differences are primarily evident in the levels of significance rather than in the interpretation of the results. Overall, the gender-stratified analyses supported the associations observed in the overall models for both depressive and anxiety symptoms.

## 4. Discussion

This study provides robust evidence on the mental health of refugees from Ukraine living in Germany based on a nationally representative sample. Furthermore, this is the first study to compare the prevalence of psychological distress among Ukrainian refugees with other migrant and refugee groups, and to identify relevant associated sociodemographic and post-migration factors.

### Prevalence of psychological distress

To account for differences in age distribution among individuals with and without experiences of migration or/and forced displacement, age-adjusted prevalences of depressive and anxiety symptoms were estimated. At approximately 21 %, refugees from Ukraine were substantially more likely to report depressive symptoms than non-migrants and non-refugee migrants. The prevalence of anxiety symptoms was also higher among refugees from Ukraine, as well as those from Afghanistan, Iran, Iraq, and Syria, than among non-migrants. 

In a non-representative cross-sectional study from Germany (n = 304), the prevalence of depressive symptoms among refugees from Ukraine was reported to be 44.7 % (PHQ-2), while the prevalence of anxiety symptoms reached 51.0 % (GAD-2), which is significantly higher than in the present representative study [31]. Overall, studies from other host countries show considerable variation regarding the prevalence of depressive (32 % – 56 %) and anxiety symptoms (18 % – 45 %) among refugees from Ukraine [16, 32–34]. This highlights that the prevalence estimates of psychological distress derived from non-probability samples may be biased and underscores the importance of robust population-based studies for obtaining reliable assessments of the mental health situation.

Furthermore, the prevalence of psychological distress varied by gender across all comparison groups, with differences being most pronounced for anxiety symptoms. Women – regardless of whether they have a history of migration or fored displacement – were affected more frequently than men. Studies on the mental health of the German-speaking adult population [35] as well as among people of selected nationalities living in Germany [36] also show that women exhibit higher prevalences of depressive and anxiety symptoms.

### Associations between sociodemographic factors and psychological distress

With regard to sociodemographic characteristics, age differences were observed among refugees from Ukraine for both depressive and anxiety symptoms. This finding is consistent with studies on the mental health of refugees from Ukraine in Poland [37, 38], which suggest that the risk of psychological distress decreases with age. This age effect has also been empirically confirmed for the German-speaking adult population [35]. The age effects on mental health can be attributed, among other things, to differing life circumstances. For instance, younger respondents are more frequently in economically and socially unstable life phases and more often report increased stress levels related to the demands of adaptation in the host country, for example in the education system or when entering the labour market. At the same time, however, they may have fewer coping resources than middle-aged or older individuals [37].

Furthermore, women from Ukraine were 1.7 times more likely to report anxiety symptoms than men. Studies from other European countries also confirm the higher vulnerability of Ukrainian women to mental disorders [37, 39, 40]. In interpreting this finding, it is important to consider that the study sample consists largely of women who fled to Germany during the first months following the outbreak of the war in 2022 and were affected by family separation. Compared with men, women’s higher vulnerability may also reflect gender-specific experiences of violence and discrimination, as well as the cumulative burden resulting from caregiving responsibilities, employment demands, and integration processes, which may further exacerbate existing inequalities [41, 42]. In contrast, neither gender nor age differences were observed among refugees from the other countries of origin considered in this study. This finding is consistent with earlier analyses of post-migration stressors and their association with the prevalence of depressive symptoms (PHQ-2) among refugees in Germany based on data from the IAB-BAMF-SOEP Refugee Survey 2016 [8].

### Associations between socioeconomic factors and psychological distress

Regarding the socioeconomic factors of education, income, and employment status, the findings indicate that these were not consistently associated with psychological distress. Specifically, only employment status was associated with depressive symptoms among refugees from Ukraine as well as from Afghanistan, Iran, Iraq, and Syria. The association between employment status and the mental health of (Ukrainian) refugees is consistent with previous studies [6, 8,15, 43]. The positive effects of employment on mental health are attributable not only to securing one’s livelihood and the resulting financial stability, but also to social relationships, daily structure, and meaningful work [44]. However, legal restrictions, discrimination, residency requirements, limited German language profciency, and the lack of recognition of qualifications represent major barrieres for refugees in securing suitable employment [45].

In addition, the analyses confirm the empirically well-established association between income and depressive symptoms among refugees from Ukraine [46], but not among refugees from Afghanistan, Iran, Iraq, and Syria. This could be attributed to the fact that, compared to refugees from other countries of origin, low income represents a more strongly subjectively perceived decline in social status among Ukrainians [47]. Analyses of the IAB-BAMF-SOEP Survey of Refugees from Ukraine in Germany show that, despite high educational attainment and extensive professional work experience, they are often employed below their original qualification level and consequently earn lower incomes [48]. This de-skilling represents an additional psychosocial burden that intensifies the psychological effects of a precarious economic situation and could thus explain the association between income and mental health. In contrast, the material situation for refugees from countries with lower wage levels (e.g., Afghanistan) may represent a relative improvement compared to their country of origin, despite precarious working conditions. This could potentially mitigate the negative association between low income and mental health.

### Associations between post-migration factors and psychological distress

Furthermore, the multivariable analyses highlight the importance of social integration – including the frequency of social contact and partnership status – for the mental health of refugees from all countries of origin, but particularly for those from Ukraine. This is consistent with previous research from other host countries, which demonstrates associations between feelings of loneliness [17, 38], social support [15], or partnership [37] and mental health. The social environment represents an important protective factor for mental health by offering both emotional and instrumental support [49]. Studies have shown that social support strengthens resilience and facilitates coping with stress arising from forced displacement as well as the challenges of adapting to and navigating a new society [50–52].

The findings further support the well-established association between experiences of discrimination and mental health among refugees from all countries of origin [53, 54], which is also evident among migrants [36]. Experiences of discrimination constitute psychosocial stressors that can exacerbate the negative effects of existing vulnerabilities (experiences of forced displacement, insecure residence status, precarious living conditions, or social marginalization) on mental health. Associations between self-reported experiences of discrimination and mental as well as physical health can be explained through approaches of ‘ecosocial theory’, which emphasizes the interplay between biological, physiological, and social processes. According to this perspective, discrimination leads to the embodiment of social inequalities, meaning that these are not only experienced socially but also become biologically embedded through chronic physiological and biological processes that have lasting effects on health [55]. Furthermore, processes of exclusion indirectly affect mental health by limiting access to societal resources – such as the labour market, education, or healthcare systems – through interpersonal, institutional, and structural forms of discrimination, thereby limiting equal participation [56]. Particularly among refugees, structural, institutional, and interpersonal forms of discrimination often intersect, leading to cumulative burdens that may reinforce one another and contribute to increased psychological vulnerability.

In addition to social integration and experiences of exclusion, housing satisfaction was associated with depressive symptoms among refugees from Ukraine. Empirical evidence confirms that housing is a key social determinant of mental health, including among refugees [57]. By contrast, no association between housing satisfaction and mental health was observed among refugees from the other countries of origin considered in this study. One possible explanation is that many of these participants had previously lived in collective accommodation during the asylum process, and therefore satisfaction with their current private housing situation may have been of lesser relevance to their mental health.

The results further indicate that poorer German language proficiency was associated with a higher risk of both depressive and anxiety symptoms, particularly among refugees from Afghanistan, Iran, Iraq, and Syria. This is consistent with an earlier study confirming that German language skills have a significant influence on feelings of loneliness as well as on psychological distress [58]. Proficiency in the majority language of the host country facilitates access to key societal resources and promotes social participation. For example, the services offered by the healthcare system are often not tailored to the diversity and linguistic variety of the population in Germany, which means that barriers to accessing psychiatric, psychotherapeutic, and psychosocial care can exacerbate health inequalities [59].

### Strengths and limitations

A major strength of this study is the representativeness and size of the sample, which enable robust conclusions about the living conditions of refugees from Ukraine in Germany and the factors associated with their mental health. Furthermore, the study is characterized by a broad range of topics covered in the collected data and the use of established survey instruments. The integration of the IAB-BAMF-SOEP Survey of Refugees into the SOEP main survey (SOEP-Core) also enables comparative analyses with refugees from other political and socio-economic contexts, as well as with the population with and without a history of migration.

Nevertheless, it is important to point out certain limitations that must be considered when interpreting the results. The cross-sectional design of the study precludes causal inference and does not allow conclusions to be drawn regarding the direction of the observed associations. Since the temporal order between the examined sociodemographic and post-migration factors and mental health cannot be clearly determined, factors such as a lack of social contacts or limited German language proficiency could be not only determinants of psychological distress (causes) but also its consequences (reverse causality). The analyses are therefore exploratory in nature and provide indications of possible associations, but not of causal mechanisms. Furthermore, the sample sizes for differentiated analyses are in some cases quite small, e.g., for determining the prevalence of depressive symptoms among Ukrainians by educational level. In addition, the validity of screening instruments for assessing mental health across a diverse population is considered controversial [60]. However, based on the representative SOEP sample, the scalar measurement invariance of the PHQ-4 could be demonstrated both for the population with and without a history of migration as well as for refugees [61]. 

### Conclusion

The present study confirms a substantially higher prevalence of psychological distress among refugees from Ukraine in Germany compared to non-migrants. The findings further suggest that health resources and risks are not associated solely with forced migration itself, but are closely linked to living conditions and the resulting opportunities for accessing societal resources in the host country. In particular, experiences of discrimination were associated with health inequalities. Furthermore, the findings point to important associations between social and socioeconomic integration and mental health among refugees from Ukraine. Targeted interventions are needed that address the mechanisms of social exclusion and offer diversity-sensitive psychotherapeutic care services to combat health inequalities. In this context, stepped care models are of particular importance, as they encompass not only individual therapeutic interventions but also measures aimed at strengthening social support networks [49]. Furthermore, longitudinal data based on representative samples are essential for monitoring the development of refugees’ mental health over the course of their integration into German society.

## Figures and Tables

**Table 1:** Sample description by sociodemographic and socioeconomic characteristics: non-migrants (n =13,210), non-refugee migrants (n = 4,889), refugees from Ukraine (n = 3,039), and refugees from other countries of origin (n = 3,011). Sources: IAB-BAMF-SOEP Survey of Refugees 2023, SOEP-Core 2023

	**Non-migrants**		**Non-refugee migrants**		**Refugees: UKR**		**Refugees:** **AFG, IRN, IRQ, SYR**	
	**n**	**% (weighted)**	**n**	**% (weighted)**	**n**	**% (weighted)**	**n**	**% (weighted)**
**Gender**								
Women	6,889	51.1	2,527	53.0	2,112	73.1	1,259	33.7
Men	6,311	48.9	2,360	47.0	927	26.9	1,749	66.3
Missing	10	-	2	-			3	-
**Age group**								
18 – 39 years	3,529	26.5	3,154	38.2	1,372	50.0	1,774	67.2
40 – 59 years	4,811	32.0	1,281	36.6	1,222	35.2	1,076	26.2
≥ 60 years	4,870	41.5	454	25.2	445	14.8	161	6.6
**Education**								
Low	941	8.6	619	19.2	271	9.0	1,850	59.1
Medium	6,512	55.7	1,538	45.5	951	30.7	694	25.2
High	5,207	35.7	2,241	35.3	1,807	60.3	389	15.7
Missing	550	-	491	-	10	-	78	-
**Income**								
< 60 % of the median	1,373	12.7	837	22.7	2,579	85.0	2,334	67.5
> 60 % of the median	11,837	87.3	4,052	77.3	460	15.0	677	32.5
**Employment status**								
Employed	8,696	61.1	1,005	69.9	641	21.7	1,126	51.3
Unemployed	4,415	38.9	3,864	30.1	2,349	78.3	1,796	48.7
Missing	99	-	20	-	49	-	89	-

UKR = Ukraine, AFG = Afghanistan, IRN = Iran, IRQ = Iraq, SYR = Syria
